# Targeting Cardiac Fibrosis with Chimeric Antigen Receptor Neutrophils Derived from Human Pluripotent Stem Cells

**DOI:** 10.64898/2026.10.11.758383

**Published:** 2026-10-13

**Authors:** Gyuhyung Jin, Shaohai Fang, Yun Chang, Kunming Shao, Huiyang Li, Yan Tan, Xiaojun Lance Lian, Qing Deng, Yun Nancy Huang, Xiaoping Bao

## Abstract

Heart failure remains a leading cause of global morbidity and mortality, with cardiac fibrosis contributing to disease progression. Following myocardial injury, activated cardiac fibroblasts orchestrate adverse extracellular matrix (ECM) remodeling, which leads to tissue stiffening and compromised contractility. Despite the clinical importance of cardiac fibrosis, current pharmacological interventions modulate disease progression without directly eliminating activated fibrogenic cells. To target these cells directly, we engineered human pluripotent stem cell (hPSC)-derived neutrophils expressing a chimeric antigen receptor (CAR) directed against fibroblast activation protein (FAP). In vitro, FAP CAR neutrophils exhibited increased reactive oxygen species-associated fluorescence during fibroblast co-culture and dose-dependent cytotoxicity in 2D cultures, while reducing fibroblast-associated fluorescence in 3D cardiac organoids without a significant reduction in cardiomyocyte-associated cardiac troponin T staining. FAP CAR neutrophils also showed increased iNOS and decreased Arginase-1 expression compared with unmodified neutrophils, consistent with a shift toward an N1-associated polarization profile. Analysis of public cardiac single-cell and single-nucleus RNA-sequencing datasets identified temporal patterns of immune-associated expression and fibroblast activation that informed treatment timing. In a murine myocardial infarction (MI) model, mice treated systemically with FAP CAR neutrophils showed higher day 7 left ventricular ejection fraction than PBS-treated mice in an exploratory comparison. Together, these findings support further evaluation of hPSC-derived FAP CAR neutrophils as an immunotherapeutic approach to cardiac fibrosis.

